# Imprinted Long Non-Coding RNAs in Mammalian Development and Disease

**DOI:** 10.3390/ijms241713647

**Published:** 2023-09-04

**Authors:** Flavio Di Michele, Isabel Chillón, Robert Feil

**Affiliations:** 1 Institute of Molecular Genetics of Montpellier (IGMM), CNRS, 1919 Route de Mende, 34093 Montpellier, France; 2 University of Montpellier, 163 Rue Auguste Broussonnet, 34090 Montpellier, France

**Keywords:** epigenetics, genomic imprinting, long non-coding RNA, transcriptional interference, chromatin repression, histone methylation, imprinting disorder

## Abstract

Imprinted genes play diverse roles in mammalian development, homeostasis, and disease. Most imprinted chromosomal domains express one or more long non-coding RNAs (lncRNAs). Several of these lncRNAs are strictly nuclear and their mono-allelic expression controls in *cis* the expression of protein-coding genes, often developmentally regulated. Some imprinted lncRNAs act in *trans* as well, controlling target gene expression elsewhere in the genome. The regulation of imprinted gene expression—including that of imprinted lncRNAs—is susceptible to stochastic and environmentally triggered epigenetic changes in the early embryo. These aberrant changes persist during subsequent development and have long-term phenotypic consequences. This review focuses on the expression and the *cis*- and *trans*-regulatory roles of imprinted lncRNAs and describes human disease syndromes associated with their perturbed expression.

## 1. Introduction

Epigenetic processes are important for the establishment and maintenance of gene expression patterns during development and after birth [1]. They bring about covalent modifications on the genome (DNA methylation) and the associated chromatin (histone modifications) that are stably maintained during somatic cell divisions. However, these epigenetic modifications are also reversible and may be influenced by environmental cues [2]. Different genetic and epigenetic mechanisms induce the mono-allelic expression of genes during development, and this critically influences their expression levels (reviewed in [3]). Genomic imprinting provides one of the best-studied examples of mono-allelic expression in mammals. During development, this epigenetic phenomenon causes a form of mono-allelic expression that is strictly dependent on the parental origin of the allele [4,5,6]. Approximately 150 protein-coding genes show imprinted expression in mice and humans, often in a tissue-specific manner. About half of the imprinted genes are expressed from their maternally inherited copy only, whereas the others are expressed only from their paternal allele [7,8].

It is because of imprinted gene expression that, in mammals, both the parental genomes are required for development and after birth [9,10,11]. Functional studies in mice have shown, for multiple imprinted genes, that their expression levels are critically important for cellular proliferation, development, and physiological processes. Other imprinted genes play key roles in brain development and behaviour [5,12]. In humans, the loss of expression, or aberrant biallelic expression, of imprinted genes can give rise to different congenital disease syndromes [13,14,15]. These pathologies are often referred to as imprinting disorders. In humans, several imprinted genes also show perturbed expression in different types of cancer, and these perturbations are thought to contribute to the process of tumourigenesis [16].

A common feature of imprinted genes is that they are organised in clusters within chromosomal domains that comprise from tens to thousands of kilobases of DNA. Each imprinted domain is controlled by a maternally, or a paternally, inherited DNA methylation imprint that is somatically maintained following fertilisation, throughout development [17,18]. These parental methylation imprints occur at essential regulatory sequence elements, thus creating a functional difference between the two parental chromosomes. The essential regulatory sequences that are marked by the germline-acquired DNA methylation imprints are called ‘imprinting control regions’ (ICRs). These differentially methylated regions (DMRs) are referred to as germline DMRs as well. Most ICRs correspond to gene promoters and are methylated on the maternal allele. During embryonic development, additional regulatory sequences acquire allelic methylation at imprinted domains, through various ICR-dependent mechanisms [6]. These somatically acquired DMRs are secondary DMRs. The importance of ICRs (germline DMRs) and secondary DMRs for imprinted gene expression has been demonstrated in multiple targeting studies in the mouse, for many of the imprinted domains [4].

In mammals, DNA methylation occurs at cytosines in the context of CpG dinucleotides (‘CpG methylation’) [19]. The parental allele-specific CpG methylation at ICRs is essential in mediating the imprinted gene expression during early development, by rendering the ICRs functionally different between the two parental chromosomes. The way in which the allelic DNA methylation at an ICR promotes mono-allelic gene expression, however, is different between the various imprinted domains [5,20].

Besides protein-coding genes, hundreds of non-coding RNAs (ncRNAs) are controlled by genomic imprinting as well [21]. In humans, for instance, approximately 7% of all the microRNAs (miRNAs) are imprinted and expressed from one of the two parental genomes only [22]. Several large clusters of small nucleolar RNAs (snoRNAs) are also imprinted. These different types of imprinted small ncRNAs play diverse roles in development and physiology, and, as for the imprinted protein-coding genes, their dosage control by imprinting is functionally important [22,23,24,25]. This review does not concern the imprinted small RNAs, however, which have been discussed in detail in recent reviews [23,26]. Instead, it focuses on the imprinted long non-coding RNAs, a class of RNAs that are emerging as essential factors in the control of protein-coding genes, with major effects on development and disease [27,28,29].

**Figure 1:**
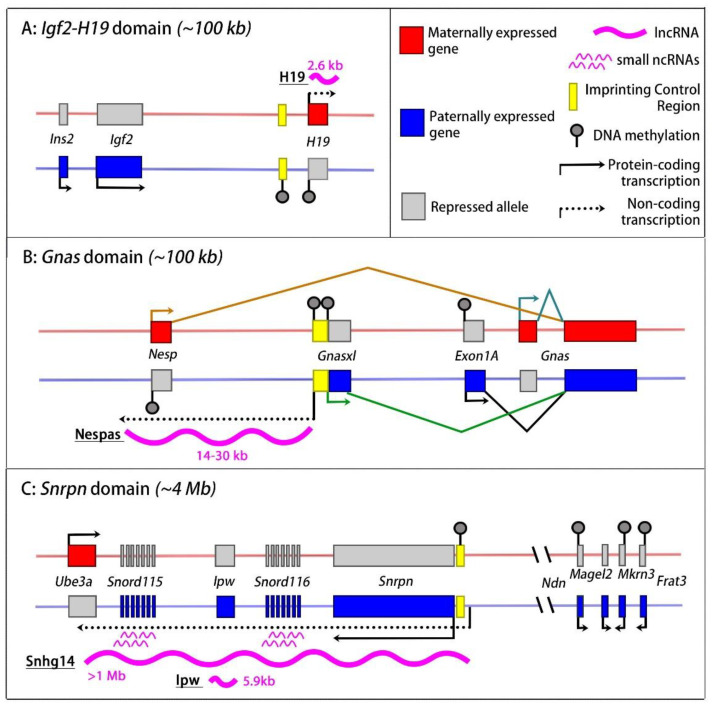
Mouse imprinted gene domains with lncRNA transcriptional or lncRNA indirect effects. (**A**) The *Igf2-H19* domain. A paternally methylated ICR (yellow rectangle) controls the maternal allele-specific expression of the H19 lncRNA (thick waved line) and the paternal allele-specific expression of *Igf2* and *Ins2*. The paternal *H19* promoter acquires DNA methylation (grey lollipop) early in development. Red and blue horizontal lines indicate the maternal and the paternal chromosome, respectively. (**B**) At the *Gnas* domain, a maternally methylated ICR mediates paternal allele-specific lncRNA expression. The lncRNA expression (of Nespas), in turn, represses the protein-coding *Nesp* gene on the paternal chromosome. (**C**) The *Snrpn* domain has an ICR that suppresses lncRNA expression on the maternal chromosome. On the paternal chromosome, transcription of Snhg14 lncRNA represses the *Ube3a* gene. The IPW lncRNA likely originates from the *Snhg14* lncRNA. On the paternal chromosome, additionally, the ICR activates the distally located *Ndn*, *Magel2*, *Mkrn3*, and *Frat3* genes, through a poorly understood process that may involve chromatin looping [63]. In the figure, the lengths of the unspliced primary lncRNAs are indicated. ICRs (yellow rectangles) have germline-acquired allelic DNA methylation (they are germline DMRs). The allelic methylation shown elsewhere in the domains is acquired during embryonic development (secondary DMRs).

Long non-coding RNAs (lncRNAs)—defined as being more than 500 nucleotides in length [27]—have attracted growing attention in the field of genomic imprinting. The first discovered lncRNAs were imprinted lncRNAs, and, already in the early days, their expression was found to control close-by protein-coding genes [17]. The first lncRNA identified in mammals—more than thirty years ago—was the imprinted H19 RNA [30]. This spliced and poly-adenylated lncRNA of 2.3-kb in size is expressed from the maternal chromosome only, in mesodermal and endodermal tissues. *H19* is part of an evolutionarily conserved chromosomal domain located on mouse chromosome 7. This imprinted domain also comprises the essential insulin-like growth factor 2 (*Igf2*), a growth-regulating gene that is expressed from the paternal chromosome only (Figure 1).

In studies on lncRNAs, it has generally been challenging to ascertain what precisely brings about the phenotypic effects of their expression [27]. For most lncRNA genes, it remains unclear whether their effects are mediated by promoter activity, by the transcription of the lncRNA, by the generated lncRNA itself, or by regulatory RNAs processed from the lncRNA. Although between 15 and 180 thousand lncRNAs are thought to be expressed by mammalian genomes—depending on the estimates (http://www.noncode.org/analysis.php, accessed on 1 September 2023)—so far, only approximately one hundred have been explored functionally [27]. Interestingly, almost all the methylation-controlled imprinted domains express one or more lncRNAs [6,12]. Several of these imprinted lncRNAs have been studied well enough to draw conclusions about their modes of action (Table 1). Much is known about how they control the expression of close-by protein-coding genes at their respective domains (*cis* effects), and some imprinted lncRNAs affect the expression of genes on other chromosomes as well. Despite considerable research efforts during the last few years, however, the in-*trans* effects remain less well understood than the *cis* actions of imprinted lncRNAs.

This review focuses on mammalian lncRNAs that are imprinted. These lncRNAs constitute only a fraction of all the known lncRNAs in mammals [27]. However, because of their allelic expression status and their involvement in development and disease, these exceptional lncRNAs have provided attractive research paradigms [31,32,33,34]. Below, we discuss how imprinted lncRNAs control chromatin organisation and gene expression, in *cis* and in *trans*, and how these functions influence development, homeostasis, and disease.

## 2. Regulatory lncRNAs at Developmental Imprinted Gene Domains

Many of the conserved imprinted domains—of which several are linked to specific imprinting disorders in humans—have been explored for their biological functions and the roles of their lncRNAs (Table 1). Although, quite logically, this review focuses on these important gene domains, it also presents data on other, less studied, imprinted loci that also express lncRNAs.

### 2.1. The Igf2-H19 Imprinted Domain

H19 was the first lncRNA discovered in mammals [30] and is expressed from the maternal genome exclusively [35]. It resides in an imprinted domain controlled by an intergenic ICR that is methylated on the paternal chromosome. This relatively small domain (~100 kb) also comprises the insulin-like growth factor 2 (*Igf2*) and insulin (*Ins*) genes, both of which are expressed from the paternal chromosome predominantly, in a tissue-specific manner (Figure 1). Whereas the expression levels of *Igf2* and *Ins* are critical for growth and homeostasis [36,37,38], initial targeting studies did not reveal marked phenotypes in *H19*-deficient animals, despite the evolutionary conservation of this lncRNA [39]. In subsequent studies, many years later, however, H19 RNA was found to reduce placental growth during foetal development. This growth-limiting effect is mediated by a miRNA that is processed from the first exon of the lncRNA, specifically in the placenta [40,41]. One target of the H19-derived miRNA is the mRNA of the growth-related IGF1 receptor gene (*Igf1r*), and this explains H19′s negative effects on placental growth [41].

In humans, the *IGF2-H19* imprinted domain (chromosome 11p15.5) is causally involved in two growth-related imprinting disorders, Beckwith-Wiedemann Syndrome (BWS, OMIM 130650) and Silver-Russell Syndrome (SRS, OMIM 1809 = 860) [42]. BWS and SRS cases that are linked to the *IGF2-H19* locus are caused by increased or decreased expression of the growth-regulating *IGF2* gene, respectively, and concordant changes in *H19* expression may contribute to the clinical aetiology of these disorders as well, through the *trans* effects of this conserved lncRNA (see also below).

### 2.2. The Igf2r Imprinted Domain

**Figure 2:**
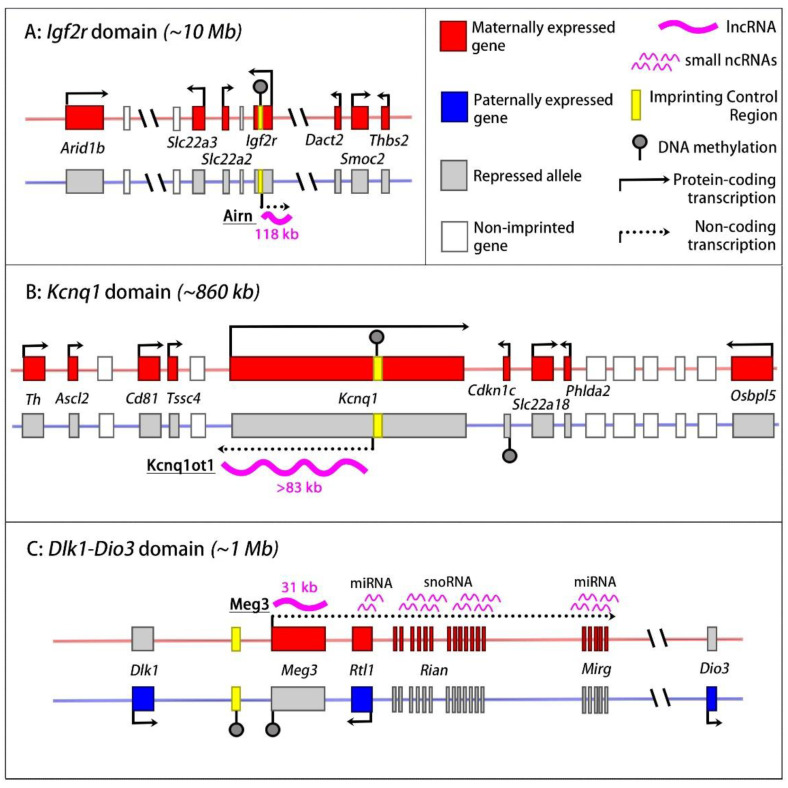
lncRNA-induced chromatin repression at imprinted gene domains in the mouse. (**A**) The *Igf2r* domain. In the placenta, the paternally expressed lncRNA *Airn* (thick waved line) induces long-range chromatin repression and silences multiple genes on the paternal chromosome (grey boxes). The ICR (yellow box) on the maternal chromosome is methylated (grey lollipop), leading to the silencing of *Airn*. In both the embryo and the placenta, *Airn* transcription also represses the paternal allele of *Igf2r*, through a transcriptional interference mechanism. The domain comprises also non-imprinted genes (white boxes). (**B**) The *Kcnq1* domain in the placenta. The paternally expressed lncRNA Kcnq1ot1 brings about repressive chromatin modifications in *cis*, which repress multiple genes on both sides of the domain. (**C**) The *Dlk1-Dio3* domain has a paternally methylated ICR. On the maternal chromosome, it activates the *Meg3-Rian-Mirg* ncRNA polycistron. Meg3 lncRNA expression, in turn, represses protein-coding genes on the maternal chromosome during stem cell differentiation. In the figure, the lncRNA lengths concern the primary, unspliced transcripts. ICRs (yellow rectangles) have allelic, germline-acquired DNA methylation (they are ‘germline DMRs’). The allelic DNA methylation shown elsewhere in the domains is acquired during embryonic development (secondary DMRs).

Another well-characterised imprinted lncRNA is Airn, at the IGF2 receptor gene (*Igf2r*) domain on mouse chromosome 17 (Figure 2). This 118-kb lncRNA is expressed from the paternal chromosome only [43]. As with the *Igf2-H19* locus, the *Igf2r* domain plays an important role in the control of foetal growth. This function is conferred mostly by the maternally expressed mannose-6-phosphate/insulin-like growth factor receptor type 2 gene (*Igf2r*), which encodes a non-functional receptor that attenuates INS/IGF signalling and thereby reduces cellular proliferation and growth [44,45]. The *Igf2r* domain is large and comprises three cation transporter genes as well, of which one (*Slc22a3*) shows expression from the maternal chromosome only, and another (*Slc22a2*), displays a strong maternal bias in its expression, in the placenta. A maternally methylated ICR within the second intron of *Igf2r* controls the imprinted expression of *Igf2r*, *Slc22a2*, and *Slc22a3*. This intragenic ICR comprises the promoter of the lncRNA Airn. Because of the ICR’s allelic DNA methylation status, *Airn* is expressed on the paternal chromosome only. Transgenic studies in mice that generated the loss of expression, or truncation, of Airn all resulted in reduced foetal growth, caused by biallelic (and hence increased) *Igf2r* expression [46,47,48]. The Airn lncRNA overlaps the *Igf2r* promoter, which brings about a transcriptional interference process, which is outlined in more detail below. In the extra-embryonic tissues, additionally, the loss of Airn lncRNA leads to the biallelic transcription of the distally located *Slc22a2* and *Slc22a3* [46,49]. The imprinted expression of these genes is due to the recruitment of lysine methyltransferases (KMTs) and the subsequent deposition of repressive histone methylation [50,51]. A recent study reported that, in the placenta, several other genes (*Arid1b*, *Park2*, *Smcc2*), across a 10-Mb region, are expressed from the maternal chromosome only. These distant imprinted genes are controlled by Airn lncRNA as well [8], which makes the *Igf2r* domain the largest known imprinted domain in mice. The latter finding also underlines that, at imprinted domains, more genes are imprinted in the trophoblast than in the embryo [8]. Combined, the long-range repressive effects of Airn attenuate placental development and, indirectly, affect foetal development as well.

The human *IGF2R* gene (on chromosome 6q25) is not imprinted, with mono-allelic expression observed in some people only [52]. However, there is expression of an Airn-like lncRNA from an intronic CpG island within *IGF2R* [53]. Although *IGF2R* is not linked to an imprinting disorder, its expression levels have been linked to the occurrence of different cancers [54,55].

### 2.3. The Gnas Imprinted Domain

Another imprinted locus at which a lncRNA mediates allelic gene expression is the *Gnas* domain on mouse chromosome 2 (Figure 1). This ~100-kb domain is important for development and endocrine regulation [12]. It comprises *Gnas*, which encodes the G protein α-subunit G_s_α, which functions downstream of G-protein-coupled receptors in response to hormones and extracellular signals. The locus also comprises the overlapping paternally expressed *Gnasxl*, which encodes a variant Gα_s_ subunit [56,57]. The domain is controlled by a maternally methylated ICR that comprises promoters leading to bi-directional transcription on the unmethylated paternal copy [58,59]. One of the generated transcripts is a lncRNA called ‘Nesp-antisense’ (Nespas), which is more than more than 14 kb in size (Table 1) and likely covering 30 kb [60,61]. On the paternal chromosome, Nespas represses in *cis* a nearby gene called *Nesp*, whose transcript overlaps *Gnas* as well [62]. This *cis*-repressive effect is similar to that of Airn at the *Igf2r* domain.

At the *Gnas* domain, a maternally methylated secondary DMR covers a promoter region that expresses a longer variant of GNAS (Exon1A variant), from the paternal allele only (Figure 1). The allelic DNA methylation at the different DMRs and the allelic *Nespas* expression are intricately linked and, together, are responsible for the allelic expression of overlapping, protein-coding transcripts from the maternal (*Nesp*, *Gnas*) and the paternal chromosome (*Gnasxl, Exon1A-Gnas*). Targeting studies in mice have shown that the expression levels of the different GNAS-like proteins have diverse metabolic and endocrine effects and influence behaviour as well [12,56].

The human *GNAS* locus on chromosome 20q13.3 shows comparable DNA methylation and gene expression patterns to those in mice and is causally involved in different forms of ‘pseudo-hypoparathyroidism’ (PHP) [64,65], an endocrine disorder characterised by reduced expression of the GNAS-like proteins. Children with PHP variably manifest bone defects with ectopic ossifications, short stature, and early-onset obesity, and their endocrine defects include resistance to parathyroid hormone (PTH) and thyroid stimulating hormone. In one form of the disease, PHP type 1b (OMIM 603233), the maternal methylation at the GNAS-Exon1A region (called GNAS A/B in humans) is lost, which leads to loss of the imprinted *GNAS* expression [64,65].

### 2.4. The Kcnq1 Domain

At the imprinted *Kcnq1* domain on mouse chromosome 7, a lncRNA called Kcnq1ot1 exerts long-range repressive effects on eight genes (Figure 2). This lncRNA has been estimated to be 83 [66], 91 [67], 121 [68], or 471 [69] kilobases in size, depending on the cell type studied. Transcription of this >83-kb lncRNA occurs from the paternal chromosome only and is driven by a maternally methylated ICR [70]. This ICR is located within an intron of an oppositely transcribed protein-coding gene called *Kcnq1*, which is important for heart function and whose mutation in humans can cause type 1 long QT syndrome (LQT1, OMIM 192500). On the paternal chromosome, Kcnq1ot1 lncRNA controls the allelic repression of the *Kcnq1* gene on the paternal chromosome [68,71]. Several other genes located further away, that do not overlap Kcnq1ot1, are repressed by the lncRNA as well, most pronouncedly in the extra-embryonic tissues, through the deposition of repressive histone modifications [50,68,72,73,74]. These genes include the cyclin-dependent kinase inhibitor 1C (*Cdkn1c*), which exerts a negative effect on cellular proliferation and growth.

The human *KCNQ1* domain is positioned adjacent to the *IGF2-H19* domain on chromosome 11p15.5 and is causally involved in the foetal overgrowth syndrome Beckwith-Wiedemann Syndrome (BWS) [42]. In this congenital disorder, early embryonic loss of methylation at the domain’s intragenic ICR induces biallelic KCNQ1OT1 expression. This, in turn, leads to the almost complete loss of *CDKN1C* expression, which causes the observed foetal overgrowth in this class of BWS. Targeting studies in mice have confirmed this phenotypic effect of the Kcnq1ot1 [70]. They have demonstrated the functional importance of Kcnq1ot1 lncRNA in chromatin repression as well, for which the conserved 5′ portion (approximately 900 bases) is particularly important [68,75].

### 2.5. The Dlk1-Dio3 Imprinted Domain

A structurally similar domain is the *Dlk1-Dio3* locus on mouse chromosome 12 [76] (Figure 2). This imprinted gene cluster is controlled by a paternally methylated ICR. Initial targeting studies in the mouse showed that the unmethylated maternal copy of this intergenic ICR controls the paternal expression of several protein-coding genes—*Dlk1*, *Rtl1*, and *Dio3*—that play diverse roles in foetal and extra-embryonic development [77]. Subsequent studies revealed that the unmethylated maternal copy of the ICR is an enhancer [78,79,80,81]. Particularly, on the maternal chromosome, the ICR activates a large polycistronic transcription unit that expresses a multitude of ncRNAs, including a lncRNA called Meg3 (also called Gtl2 [82]), twenty-two C/D-box snoRNAs (*Rian* locus), and some fifty miRNAs (*Mirg* locus) [77,78,81,83].

The maternal expression of the ncRNA polycistron, in turn, is essential for the allelic repression of the domain’s protein-coding genes. Targeting studies in cells and animals have suggested that it is the Meg3 lncRNA that represses the protein-coding genes on the maternal chromosome [84]. Concordantly, Meg3 is strictly nuclear and is retained at the imprinted locus in embryonic cells [84]. The mechanism through which this 31-kb lncRNA represses close-by protein-coding genes will be presented below.

The organisation of the *Dlk1-Dio3* imprinted locus—and its paternally methylated ICR—is conserved amongst mammals [76,85]. In humans, the locus maps to chromosome 14q32. Epimutations and microdeletions that affect the paternally methylated ICR, or the promoter of the *MEG3* polycistron, are causally involved in two congenital imprinting disorders: Temple Syndrome (TS14, OMIM 616222) and Kagami-Ogata Syndrome (KOS14, OMIM 608149) [83,86,87,88]. TS14 is characterised by growth retardation, premature puberty, and obesity and its most common cause is maternal uniparental disomy (MatUPD14) of chromosome 14q32, where the *DLK1-DIO3* domain resides [89]. KOS14, in contrast, often caused by PatUPD14, is characterised by skeletal dysmorphism, placentomegaly, and polyhydramnios [89]. The two imprinting disorders have in common that the activity of the MEG3 ncRNA polycistron is either fully ablated (KOS14) or becomes biallelic (TS14) [89]. As in mice, this observation evoked a putative *cis*-regulatory role for the lncRNA MEG3. In patients, however, observed methylation changes can be mosaic and can involve multiple imprinted loci [13,90], which has complicated the drawing of mechanistic conclusions about the human locus.

### 2.6. The Snrpn Domain

Another domain that is causally involved in imprinting disorders is the *SNRPN* gene cluster on human chromosome 15q11-13 (mouse chromosome 7, Figure 1) [91]. This ~4-Mb domain is causally involved in two different neurodevelopmental/behavioural syndromes: Prader-Willi Syndrome (PWS, OMIM 176270) and Angelman Syndrome (AS, OMIM 105830) [92]. PWS patients show developmental delays with poor suckling and hypogonadism and develop behavioural impairments including hyperphagia during childhood, leading to severe obesity. In AS, there is a developmental delay as well, with microcephaly and severe mental disability, limited speech abilities, and sleeping problems [93]. This domain is conserved in mice (Figure 1). It has a maternally methylated ICR, located at the 5′ side of *Snrpn*, an imprinted gene that encodes an RNA-binding protein involved in RNA processing. Besides the allelic expression of *Snrpn*—from the paternal chromosome only—the ICR region also drives the allelic transcription of a lncRNA called *Sngh14* [94]. *Sngh14* transcription extends over more than one megabase, across a region comprising two clusters of snoRNAs (*Snord116* and *Snord115*) genes and a small internal lncRNA called IPW (‘imprinted gene in Prader-Willi Syndrome region’). Importantly, the 3′ end of the Sngh14 transcription overlaps the gene *Ube3a* (ubiquitin-protein ligase E3A). Through a transcriptional interference mechanism presented in more detail below, this mechanism leads to *Ube3a* silencing on the paternal chromosome [95]. In AS, there is aberrant biallelic expression of *SNGH14*, and, as a consequence, there is no longer any expression of *UBE3A*. In PWS, conversely, there is the loss of *SNGH14* expression and the loss of expression of several other paternally expressed genes located at the distal side of the domain. Functional studies in the mouse confirmed that *Sngh14* expression regulates the imprinted expression of *Ube3a* and revealed the role of transcriptional overlap in this process [95,96,97].

## 3. *Cis*-Regulatory Effects of lncRNAs at Imprinted Domains

**Figure 3:**
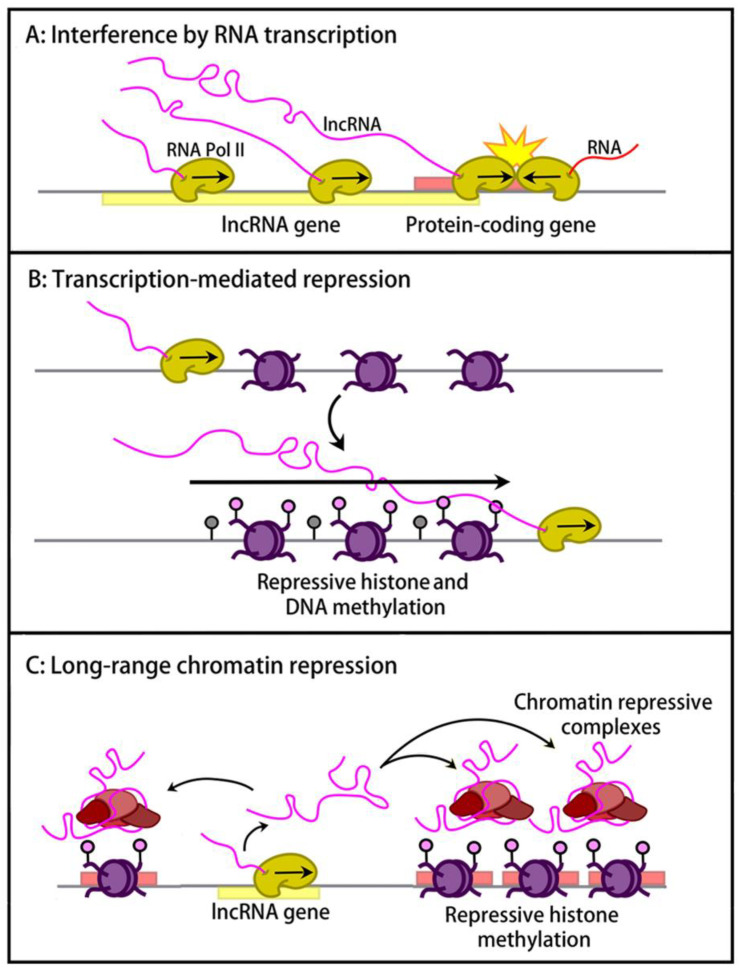
Models of how lncRNAs regulate gene expression in *cis*. (**A**) lncRNA transcription can interfere with that of an overlapping protein-coding gene, in case the overlapping gene is transcribed in the opposite direction. In the shown example, there is collision of RNA Pol II complexes, which prevents the formation of full-length transcripts from the protein-coding gene. (**B**) lncRNA transcription across promoters can lead to their repression, by preventing accessibility to RNA Pol II and by mediating repressive histone and DNA methylation. (**C**) lncRNAs may have long-range chromatin-repressive effects that involve the recruitment of specific lysine methyltransferases (KMTs) and of Polycomb repressive complexes (PRCs).

For a growing number of lncRNAs, *cis* effects on close-by protein-coding genes have been explored [27]. At imprinted domains, the lncRNAs are expressed in a mono-allelic manner and effects in *cis* are therefore expected to be allele-specific. It is because of their putative roles in imprinted gene expression that imprinted lncRNAs have attracted considerable attention. Different mechanistic models have emerged from the studies so far. These are broadly based on whether it is the lncRNA transcription that exerts the in-*cis* effect, or whether it is the lncRNA itself that is functionally important (Figure 3). It is interesting to note that many non-imprinted lncRNA genes exert a positive, enhancer-like effect on the expression of close-by genes [98,99,100,101,102]. All imprinted lncRNA genes studied so far seem to have repressive functions. This is one other aspect that distinguishes this group of lncRNAs.

### 3.1. lncRNA-Transcription-Mediated Interference and Chromatin Repression

As concerns the role of lncRNA transcription, one mechanism that has emerged is interference with an overlapping gene transcribed in the opposite direction (Figure 3A). At the *Snrpn* imprinted domain, in neurons, the transcription of the paternally expressed *Sngh14* overlaps a small protein-coding gene, *Ube3a* (ubiquitin-protein ligase E3A), which is transcribed in the opposite orientation [103]. The lack of *Ube3a* transcripts from the paternal chromosome is thought to arise through the stalling of RNA polymerase II (RNA Pol II) complexes that encounter *Sngh14*-transcribing RNA Pol II complexes moving in the opposite direction. Evidence for this mechanism came from mice that no longer expressed the lncRNA or that showed the expression of truncated forms of *Sngh14* no longer overlapping *Ube3a*, which showed biallelic *Ube3a* expression [95].

In PWS patients, similarly, the pathological loss of *SNGH14* expression correlates with the biallelic expression of *UBE3A* [104]. In human cultured cells, ectopically expressed antisense oligonucleotides directed to the lncRNA led to the activation of the normally silent paternal *UBE3A* gene. This experimental approach provided a strategy to alleviate the clinical symptoms of Angelman Syndrome (AS), a complex neuro-behavioural disease that is caused by the loss of *UBE3A* expression in the brain [96,104,105], and it is currently under a phase 1 clinical trial (https://www.roche.com/solutions/pipeline/, accessed on 1 September 2023). Upstream of the region of overlap with *Ube3a*, the *Sngh14* transcription unit comprises a cluster of approximately 75 snoRNA sequences (the *Snord115* locus, Figure 1C). A recent CRISPR-based approach used an adenoviral vector to express a guide RNA against this multi-copy sequence, as well as a short Cas9 protein variant. In an AS syndrome mouse model, this approach led to the long-lasting loss of *Sngh14* expression in the brain and, consequently, to the efficient reactivation of the silent *Ube3a* gene, possibly because of the concomitant silencing of the primary Sngh14 transcript [106].

When lncRNA transcription moves across the promoter of a flanking gene, the acquisition of repressive chromatin modifications can occur (Figure 3B). This seems to be the scenario at the imprinted *Gnas* locus (Figure 1), where Nespas lncRNA overlaps the promoter of the *Nesp* gene [56]. lncRNA truncations and other targeting events in the mouse have shown that the loss of Nespas transcription leads to the activation of the normally silent paternal *Nesp* gene [60,107]. The lncRNA-mediated repression of *Nesp* occurs early in development and involves the acquisition of DNA and histone methylation [107,108]. How precisely the process works is unclear. However, it is known from epigenomic and functional studies that progressive RNA Pol II complexes recruit SETD2, a lysine methyltransferase that brings about lysine-36 trimethylation on histone H3 (H3K36me3). The H3K36me3 acquired along the transcribed region induces the specific recruitment of the DNA methyltransferase DNMT3B, which gives CpG methylation [109,110]. This scenario explains why, at highly expressed genes, DNA methylation levels are relatively high in the gene body. At *Nesp*, in addition, there is the acquisition of repressive H3 lysine-9 trimethylation (H3K9me3), involving a yet unknown mechanism.

A similar mechanism has emerged at the imprinted *Igf2r* domain (Figure 2). Airn, the 118-kb lncRNA of this domain, is transcribed in the opposite direction to *Igf2r* and overlaps its promoter [17]. Different studies have suggested that, in the early embryo, Airn transcription prevents the recruitment of RNA Pol II complexes to the *Igf2r* promoter. Upon the differentiation of embryonic stem cells, in addition, there is the acquisition of DNA methylation and H3K9me3. Although these covalent modifications are sufficient to prevent RNA Pol II recruitment to the promoter in differentiated cells, the expression of the lncRNA is initially required for the maintenance of *Igf2r* repression on the paternal chromosome [46,47,111,112].

At other imprinted domains, lncRNA transcription prevents the expression of overlapping promoters during development as well. At the mouse *Dlk1-Dio3* domain, for instance, the maternally expressed Meg3 ncRNA polycistron overlaps *Rtl1* (Retrotransposon-like 1), a gene that is important in placental and muscle development. *Rtl1* is expressed on the paternal chromosome only. Its expression becomes biallelic in cells in which the overlapping Meg3 ncRNA polycistron is no longer transcribed, suggesting a transcriptional interference mechanism.

At the *Gpr1-Zdbf2* domain on mouse chromosome 1, an oocyte-acquired DNA methylation imprint within the *Gpr1* gene (G-protein-coupled receptor 1) brings about the imprinted expression [113,114]. On the unmethylated paternal allele of this ICR, promoter sequences express a long intergenic RNA isoform of Zdbf2 during pre-implantation development and early gastrulation. This 114-kb transcript is called Zdbf2linc (Zdbf2-long intergenic non-coding) [113], or Liz (‘long isoform of Zdbf2′) [115], and overlaps the *Zdbf2* transcription factor gene. The transient expression of *Zdbf2/Liz* during early development brings about repressive DNA methylation at a CpG island located upstream of *Zdbf2*. This somatically acquired allelic methylation imprint is stably maintained subsequently and controls the paternal allele-specific expression of *Zdbf2,* which occurs later in mouse development [115,116,117]. The epigenetic lncRNA-linked regulation of this imprinted domain is in part conserved in humans [118].

In humans, the imprinted *DIRAS3* gene (also known as *NOEY1* or *ARH1*) on chromosome 1p31 encodes a protein of the RAS superfamily of GTPases. This tumour suppressor gene is controlled by a maternally methylated ICR and shows expression from the paternal chromosome predominantly [119,120]. This imprinted gene is located within an intron of a large lncRNA called GNG12-AS1. GNG12-AS1 is a stable nuclear lncRNA, detected by RNA FISH at its site of transcription [119]. In different cancers, there is altered GNG12-AS1 expression. Studies with siRNAs that silenced GNG12-AS1 showed that reduced lncRNA expression causes the concomitant upregulation of *DIRAS3* mRNA levels, suggestive of an interference mechanism through which lncRNA transcription controls the expression of the protein-coding gene [121].

### 3.2. lncRNA-Mediated Long-Range Chromatin Repression

In another mechanism observed at several large chromosomal domains, lncRNAs can promote repressive chromatin modifications and gene repression (Figure 3C). Since, in these *cis* effects, there is the repression of genes that do not overlap the lncRNA, the lncRNAs themselves must be involved. In agreement with this hypothesis, these lncRNAs are all nuclear and accumulate in *cis* onto their imprinted domains. This mode of long-range repression shows similarities with X inactivation in female cells, a developmental process in which the lncRNA Xist coats the X chromosome and facilitates the recruitment of repressive chromatin complexes [122].

A *cis* chromatin-repressive role has been reported for the lncRNAs Kcnq1ot1 (*Kcnq1* domain), Airn (*Igf2r* domain), and Meg3 (*Dlk1-Dio3* domain) (Figure 2). These lncRNAs are all strictly nuclear and show *cis* accumulation onto their respective imprinted domains, on the parental chromosome from which they are transcribed. Their allelic *cis* retention is relatively stable and persists several hours after the inhibition of RNA Pol II, suggestive of factors that locally stabilise these lncRNAs [50,74,78,123].

Amongst other interactions, Kcnq1ot1, Airn, and Meg3 associate with components of chromatin regulatory complexes, and this interaction may contribute to the *cis* retention of these lncRNAs. Protein–lncRNA interactions have been studied most extensively for Kcnq1ot1. In the preimplantation embryo and in the placenta, this >83-kb RNA interacts with EHMT2 (also called G9A), a lysine methyltransferase (KMT) that brings about H3 lysine-9 dimethylation (H3K9me2), and with components of the polycomb repressive complexes 1 and 2 (PRC1 and PRC2), which mediate H2A lysine-119 mono-ubiquitination (H2AK119u1 and H3K27me3, respectively) [50,68,72,74,75,81,124]. In the placenta, there is the enrichment of these repressive modifications along most of the imprinted domain, on the Kcnq1ot1-expressing paternal chromosome [50,72,73]. Functional studies on EHMT2 and on EED, an essential component of the PRC2 complex, have shown the importance of these lncRNA-mediated chromatin modifications in the allelic repression of genes in the trophoblast and in embryonic stem cells [50,68,74,81,125,126]. There remains the question as to why the Kcnq1ot1-mediated chromatin repression involves many more genes in the extra-embryonic lineages than in the embryo proper (Figure 2). Possibly, trophoblast-enriched factors interact with the lncRNA to facilitate the recruitment and/or the activity of the PRC complexes and of the KMTs. One such factor could be the RNA-interacting nuclear matrix protein hnRNPK, which is essential for the PRC2-mediated H3K27me3 deposition along the domain in trophoblast stem cells [50,127].

The mechanism of action of Meg3 at the *Dlk1-Dio3* locus could be similar to that of Kcnq1ot1 at the *Kcnq1* domain (Figure 2). In the developing embryo, the maternal Meg3 expression is required for the repression in *cis* of the developmental *Dlk1* gene [84]. Earlier studies have shown that this lncRNA interacts with PRC2 components EZH2 and JARID2, and a recent paper suggests it interacts with hnRNPK as well [50,81,124,128]. PRC2 complexes are required for the imprinted gene expression at this locus, and this process depends on the level of expression of Meg3 lncRNA as well [81,84,124,129]. Which part(s) of the lncRNA is involved is not known. However, its *cis* retention onto the locus also includes unspliced RNAs, and both intronic and exonic sequences within the 32-kb primary transcript seem to interact with the PRC2 components EZH2 and JARID2 [84,124,128].

Airn RNA at the mouse *Igf2r* domain—for which, above, we have described its transcriptional interference effects—has a chromatin-repressive role as well. This long-range effect controls the allelic repression of multiple distal genes (*Slc22a2, Slc22a3, Arid1b, Park2, Smcc2*) and is observed in the extra-embryonic lineages only [8] (Figure 2). Recent targeting studies in the mouse showed that a truncated form of Airn lncRNA is no longer able to repress these genes in *cis*, whereas increased Airn expression led to stronger gene repression in *cis* [8,50,51]. Airn-induced chromatin repression involves H3K27me3 and H2AK119u1, controlled by PRC2 and PRC1 complexes, respectively [49,50], and also H3K9me2/3, brought about by the lncRNA-mediated recruitment of the KMT EHMT2 [51,126]. Airn lncRNA also interacts with hnRNPK and this interaction may enhance the allelic recruitment of PRC complexes onto the locus in trophoblast cells [50].

In conclusion, Kcnq1ot1, Airn, and Meg3 display similar repressive effects in *cis* and control the recruitment and/or the activity of KMTs and PRC complexes. Other lncRNAs, at other imprinted gene domains, might induce long-range chromatin repression in a similar manner as well. For instance, at the placental transcription factor *Tfpi2* gene on mouse chromosome 6, there is a requirement for the PRC2 complex and for the KMT EHMT2 for the gene’s allelic repression on the paternal chromosome. *Tfpi2* is part of a large imprinted domain (1.8 Mb) also comprising the *Peg8* gene, which is under the control of a maternally methylated ICR [130]. While not known yet, it would be interesting to explore whether a lncRNA is responsible for the long-range chromatin repression at this domain.

### 3.3. Putative Structural Roles in cis of Imprinted lncRNAs

Could imprinted lncRNAs also have chromatin structural effects? Recent reviews discuss this possibility and present known links between lncRNA expression and long-range chromatin structural interactions [131,132]. For instance, one way that the transcription of lncRNAs could impact the chromatin structure is by keeping binding sites for CTCF (‘CCCTC-binding factor’) non-methylated, thus ensuring the continued binding of this chromatin structural protein. The CTCF protein comprises an RNA-binding motif essential for CTCF recruitment to many of its genomic binding sites [133,134]. Intriguingly, several imprinted lncRNAs are transcribed across, or close to, CTCF-binding sites. Their allelic expression could thus influence the allelic binding or activity of CTCF and, hence, influence long-range structural interactions within the imprinted domain. In addition, as discussed above, several imprinted lncRNAs interact with PRC complexes and other chromatin-associated factors. Such interactions could locally give rise to lncRNA–protein aggregates—possibly involving liquid–liquid phase separation—thus altering the physical proximity between different regions within an imprinted domain [131,135]. Potential links between imprinted lncRNAs and chromatin structure would be interesting to explore further in the future.

## 4. Emerging *trans*-Regulatory Roles

Imprinting disorders are syndromic diseases, each defined by combinations of clinical phenotypes that manifest at different frequencies. Each of these diseases is predominantly linked to dysregulated gene expression at one imprinted domain. Despite the association of imprinting disorders with individual genomic domains, intriguingly, there is considerable clinical overlap between the different imprinting disorders [13,14]. This observation suggests the possibility that genes at different imprinted domains could act in common pathways. One example is provided by the INS/IGF pathway. This growth-regulating signalling pathway comprises the disease-associated imprinted genes *IGF2*, *IGF2R*, and *INS*, and the growth factor receptor-binding protein encoding *GRB10*, an imprinted gene for which it is unclear whether it is involved in imprinting disorders [136,137]. Different other biological functions, including nutrient and ion transport, are controlled by multiple imprinted genes at different domains as well, which underlines that imprinted genes are involved in common biological functions [12].

The clinical overlap between different imprinting disorders evokes possible mechanistic links between different imprinted domains. A regulatory protein produced by one imprinted domain, for instance, could influence the expression of genes at another imprinted domain [138]. One example is provided by *Plagl1* (also called *Zac1*) on mouse chromosome 10, which encodes a transcription factor that controls the expression of many other genes, including *Igf2* and *H19*, and the imprinted *Kcnq1ot1* lncRNA gene [139]. In humans, the loss of *PLAGL1* expression causes ‘transient neonatal diabetes mellitus’ (TNDM, OMIM 601410), an imprinting disorder characterised by intra-uterine growth restriction, similarly to what is observed in Silver-Russell Syndrome (SRS), an ID most often caused by reduced *IGF2* expression.

### 4.1. lncRNAs That Influence Other Imprinted Domains

**Figure 4:**
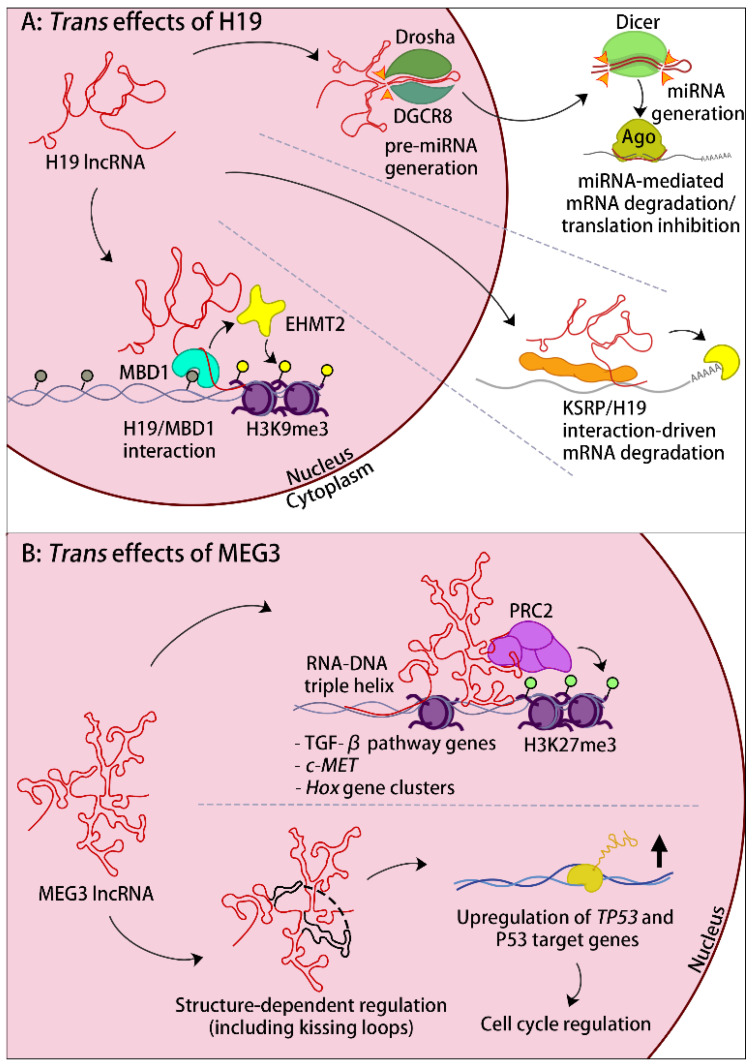
Examples of imprinted lncRNAs that have *trans*-regulatory roles. (**A**) In the nucleus, H19 lncRNA associates with the methyl-CpG-binding protein MBD1 at specific genomic loci. This enhances the recruitment of EHMT2 (also known as G9A), leading to repressive H3K9me3. H19 is mostly in the cytoplasm, where its interaction with the RNA-binding protein KSRP induces the degradation of specific mRNAs. H19 lncRNA also serves as a miRNA host. The resulting mature miRNA is loaded onto Argonaute (Ago) and, through the recognition of specific seed sequences, induces the degradation or translational inhibition of specific mRNAs. (**B**) MEG3 lncRNA forms RNA–DNA triplex structures through a specific GA-rich sequence motif. This interaction allows the targeting of several TGFβ pathway genes and of the proto-oncogene *c-MET*. At these loci, and at *Hox* gene clusters, the lncRNA associates with the PRC2 components, which locally enhances repressive H3K27me3. In cancer cells, overexpression of MEG3 activates the P53 pathway and enhances the expression of a specific subset of P53 target genes. This process requires two structural domains within the lncRNA that form interacting loops. The thick vertical arrow indicates increased gene expression.

There is growing awareness that imprinted lncRNAs could have regulatory functions in *trans* and could thus influence gene expression at other imprinted domains, possibly in the context of imprinted gene networks [25,139,140]. Thus far, however, only a few studies have provided evidence for such *trans* roles [138]. In one study, the overexpression of the *H19* gene in adult mice led to the reduced expression of *Igf2* and also the altered expression of five other imprinted genes, located on different chromosomes [141]. Mechanistically, the H19 RNA was found to interact with a methyl-CpG-binding protein called MBD1, which correlated with the enhanced binding of MBD1 to the DMRs associated with the perturbed imprinted genes [142]. The lncRNA-MBD1 association in turn enhanced the local recruitment of EHMT2 (also called G9A), a KMT that brings about repressive histone H3 lysine-9 methylation. Combined, these data suggest that H19 lncRNA controls imprinted genes through its association with MBD1 and through its recruitment to multiple imprinted gene loci (Figure 4A). What could determine the specificity of this process remains unknown.

A similar role was reported for the lncRNA IPW at the imprinted *SNRPN* domain (Figure 1). This paternally expressed lncRNA originates from the large SNGH14 non-coding RNA transcription unit. In induced pluripotent stem cell (iPSC) lines generated from PWS patients that did not express IPW, there was enhanced expression of MEG3 and of the other maternally expressed ncRNAs of the *DLK1-DIO3* imprinted domain [143]. MEG3 expression became normal again in these pluripotent cells with the overexpression of the IPW lncRNA. Mechanistically, this lncRNA has been proposed to influence the activity of the KMT EHMT2, and to thereby enhance H3K9me3 levels at the *MEG3* promoter [143]. This example provides another link within the ‘imprinted gene network’ [139,140], mediated through the specific *trans* effects of imprinted lncRNAs.

In addition, the lncRNA MEG3 could regulate imprinted genes elsewhere in the genome. Temple Syndrome (TS14) in humans is associated with aberrant biallelic expression of the MEG3 ncRNA polycistron at the *DLK1-DIO3* domain. As part of its clinical spectrum, observed in a subgroup of TS14 patients, there is reduced foetal growth. In recent studies on patient-derived serum and fibroblasts, it was found reduced levels of expression of the growth-regulatory *IGF2* gene, without apparent epigenetic changes at the *IGF2-H19* locus [86]. To explore whether MEG3 lncRNA could indeed influence the levels of *IGF2* expression, the authors reduced MEG3 expression in a primary fibroblast line using a siRNA approach. They found that the lncRNA downregulation led to a moderate increase in *IGF2* mRNA levels [86]. How, precisely, MEG3 lncRNA influences the expression of *IGF2*, and whether this observation indicates a direct or an indirect effect, remains to be determined.

The observed *trans* effects of H19 and IPW, and possibly of MEG3, on imprinted genes on other chromosomes require confirmation in follow-up research. It remains also unclear what directs these lncRNAs to their imprinted targets elsewhere in the genome. Whether this process is conferred by specific RNA sequence motifs, or by RNA structural features, would be interesting to explore. In addition, one might expect these imprinted lncRNAs to influence the expression of non-imprinted genes as well. The latter has been explored extensively for Meg3, in cancer cells and during embryonic development (Figure 4B).

### 4.2. Non-Imprinted trans Targets of Imprinted lncRNAs

EZH2, the catalytic H3K27 methyltransferase of the PRC2 complex, interacts with approximately 20% of all lncRNAs [128,144]. Amongst the interacting lncRNAs is Meg3, and multiple sites of binding were mapped to the first third of the RNA, with particular importance of exon 3 [145]. As with many other lncRNAs, Meg3 also interacts with JARID2 [124], a developmentally regulated cofactor that facilitates PRC2 recruitment to target genes during development.

In an RNA-FISH study on breast cancer cells that showed considerable MEG3 expression (MEG3 is usually silenced in cancer tissues [146]), the lncRNA formed many nuclear accumulation foci, suggesting that there could be interactions with multiple *trans* targets [145]. Using an RNA hybridisation capture assay, several TGFβ pathway genes emerged as a common target with the PCR2 complex EZH2 component. MEG3 lncRNA knockdown reduced the H3K27me3 levels at these genes, and the reduced Meg3 expression correlated with increased expression of TGFβ pathway genes [145]. Further studies on cancer cells pinpointed a common 10-bp AG repeat in the promoter-distal regions of the MEG3-regulated target genes. The same GA repeat is found at the 5′ extremity of the lncRNA itself. Therefore, this sequence was suggested to form DNA–RNA triplex structures, and these were detected at some of the target genes. The importance of this GA repeat was confirmed by cloning it into another lncRNA, KHSP1, finding that the modified lncRNA was tethered to the TGFBR1 MEG3 target gene [147]. Combined, these studies evoke an RNA-sequence-driven mechanism that tethers and stabilises the MEG3 lncRNA onto specific target genes through the formation of RNA–DNA triplexes, provoking local chromatin repression (Figure 4B).

In a study on pancreatic neuroendocrine tumour cells, MEG3 lncRNA was similarly found to interact with a gene encoding the oncogenic hepatocyte growth factor receptor c-MET—most likely through the formation of RNA–DNA triplex structures. In this study also, the data suggest locus-specific PRC2-mediated chromatin repression [148]. A mechanism of gene recognition in *trans* through RNA–DNA triplex formation has been suggested for Kcnq1ot1 as well [149] (and see below), and for several non-imprinted lncRNAs [150,151].

During development, Meg3 becomes highly expressed in the brain, particularly in neurons [152,153], and shows a nuclear, multifocal accumulation pattern. A recent biochemical study on motor neurons showed that the Meg3 lncRNA facilitates the interaction of the PRC2 complex with JARID2 [152]. In these post-mitotic neural cells, Meg3 knockdown through an shRNA approach, and maternal deletion of the domain’s ICR that controlled Meg3 expression, led to a marked decrease in H3K27me3 at some six hundred gene loci, including the caudal *Hox* genes. Concordantly, mouse embryos with maternal

ICR deletion showed aberrant *Hox* gene expression and peripheral innervation defects [152]. These interesting findings in mice are relevant for our understanding of Kagami-Ogata Syndrome (KOS14), which is caused by the loss of expression of the MEG3 ncRNA polycistron and is characterised by dysmorphic growth and skeletal defects [89]. Interestingly, the MEG3-facilitated PRC2 complex interaction with JARID2 has also been described in humans, suggesting a conserved gene expression molecular mechanism [124]. In this respect, Meg3′s repressive *trans* effects in cancer cells and neurons seem mechanistically similar to its effect in *cis* at the *Dlk1-Dio3* domain, where it enhances the local levels of H3K27me3 and represses gene expression as well [84].

In different types of cancer, including pituitary adenomas, ovarian cancer, and pancreatic neuroendocrine tumours, among others, there is reduced expression of MEG3 [146,148,154,155]. Interestingly, the loss of this lncRNA was found to correlate with the reduced expression of many different genes, including *TP53* (encoding P53) and P53 target genes [145,156]. This finding suggested that MEG3 lncRNA could induce gene expression as well. Evidence for this mechanism has come from studies in cancer cells, in which transgenic MEG3 overexpression enhanced the expression of reporter constructs that comprised P53 response elements [157]. In this study, MEG3 overexpression also stimulated the expression of the growth differentiation factor 15 (*GDF15*), by enhancing the binding of P53 to its promoter [157].

To assess which part(s) of the MEG3 RNA could be functionally important, a recent study determined the secondary structure and imaged the compact tertiary topology of this lncRNA. They found that, within a conserved part of MEG3, two structural motifs interact with each other, forming long-range tertiary interactions known as pseudoknots or ‘kissing loops’. Significantly, single nucleotide mutations that disrupted this structural feature strongly affected the stimulation of the P53 reporter genes by MEG3 [158]. This original structural study provides strong evidence for the regulation of the P53 pathway by MEG3, in a process that involves conserved tertiary structures within the lncRNA (Figure 4B).

Which other imprinted lncRNAs might control non-imprinted genes in *trans* is unknown. However, a recent study suggests that this could be the case for Kcnq1ot1. In human HEK293T cancer cells, KCNQ1OT1 expression influences the abundance of H3K9me3 foci in the nucleus, and deletion studies show that this effect is conferred by a repeat-rich region at the 3′ part of the lncRNA [149]. In these cells, KCNQ1OT1 was also found to bind to HP1α, a heterochromatin protein that interacts with H3K9me3. By performing CHIRP-seq, an RNA hybridisation assay that precipitates the genomic chromatin sites bound to the bait RNA, the authors identified evolutionarily young transposons as the main targets of the KCNQ1OT1 RNA in cancer cells. Their recognition seems to be mediated by repeat elements within the 3′ part of the lncRNA, with the formation of RNA–dsDNA triplex structures. To test whether KCNQ1OT1 RNA protects against the activation of transposons, the authors explored the importance of the repeat-rich region and found that the deletion of these repeats decreased DNA methylation and increased the transposition of LINE-1 elements [149]. Further studies are required to explore whether this *trans* effect is seen in primary cells as well, and to what extent it is conserved. Since the 3′ part of human KCNQ1OT1 is poorly conserved compared to the first half of the lncRNA, the reported repressive effects on transposons may not be conserved in mice.

H19 is different compared to other imprinted lncRNAs in that it is mostly cytoplasmic [30]. Different studies have addressed its role in the cytoplasm [159,160]. H19 is highly expressed during myogenic differentiation and its depletion enhances muscle regeneration [161]. In undifferentiated mesenchymal C2C12 cells, the RNA-binding protein ‘K homology-type splicing regulatory protein’ (KSRP) was shown to interact with H19 RNA. This cytoplasmic protein–RNA interaction was shown to enhance the action of KSRP in mRNA decay, with the increased destabilisation of labile transcripts including myogenin mRNA. The combined studies suggest that H19 provides a scaffold that facilitates the interaction of KSRP with myogenin and other labile transcripts and thereby influences myogenesis [159].

As discussed above, several imprinted lncRNAs, including H19 [41] and Nespas [162], are a reservoir of miRNAs. After their processing, the produced miRNAs reduce the stability or translation of specific mRNAs in the cytoplasm (reviewed in [31]). Although, functionally, there is no involvement of the lncRNAs themselves, the biological outcomes are relevant nevertheless, with specific effects on mRNAs expressed elsewhere in the genome.

Another way in which lncRNAs can affect development and disease is by acting as competing endogenous RNAs (ceRNAs) for small regulatory RNAs—for instance, as sponges for microRNAs [163]. This emerging RNA function has not yet been explored in a structured manner for imprinted lncRNAs. Most studies so far have been on cancer cells and provide correlations rather than experimental proof. Nevertheless, putative mechanisms of miRNA control have emerged from the many recent studies, particularly for the lncRNA H19 [164], whose potential role in controlling miRNAs has been investigated in different types of cancer, including breast and gastric cancer [165,166]. In the context of development and physiology, interestingly, H19 lncRNA expression was reported to modulate the functions of Let7 microRNAs [160,167]. Significantly, the deletion of the matching sequence motifs from *H19*—while keeping the rest of the lncRNA intact—was found to affect cardiac physiology in a recent in vivo mouse study [168].

## 5. Perspectives

Many exciting insights have emerged regarding imprinted lncRNAs and how these control close-by genes. During the last few years, evidence has also been obtained for the diverse *trans* roles of imprinted lncRNAs. These insights have been important for our understanding of the complex aetiology of imprinting disorders. For many imprinted lncRNAs, however, it remains unclear whether or not they control gene expression. It remains complicated to determine whether a lncRNA is important because of its transcription—for instance, through transcriptional interference—or whether the RNA itself mediates the observed effects. Deletion of the lncRNA gene is not informative enough, because this approach ablates both transcription and the RNA. As shown for Meg3 and Kcnq1ot1, investigation of the effects of small deletions and of specific structural changes is a more promising way forward. Admittedly, the mechanistic understanding of imprinted lncRNAs is lagging behind that of Xist, the lncRNA involved in X-chromosome inactivation in female embryos [122]. In part, this is due to a lack of knowledge of the RNA-interacting proteins in specific tissues and cells. It is also challenging that lncRNA genes often express multiple splicing isoforms. Different isoforms may acquire different three-dimensional structural organisations; they may interact with different factors and may have different functions altogether. This complexity needs to be taken into account. Despite the development of powerful novel technologies, including RNA hybridisation capture [169,170], it also remains challenging to pinpoint the *trans* targets of lncRNAs and to discern between direct and indirect transcriptional effects. Here, studies into the structure of a lncRNA, and, linked to this, into interactions with specific protein factors, may provide helpful insights.

It will be interesting to explore further to what extent lncRNAs are perturbed in their expression in patients with imprinting disorders. Could shifts in specific isoforms, or changes in post-transcriptional lncRNA modifications, be linked to human diseases as well? With the recent identification of imprinted lncRNA target genes, this needs to be taken on board in future studies as well, particularly in case these are potentially relevant for the clinical phenotype.

To better understand lncRNA expression in human disease, one needs to consider to what extent the mechanisms detected in mouse studies are evolutionarily conserved in humans. For instance, the *Igf2r* gene is clearly imprinted in the mouse but imprinting is lost in primates, with the detection of biallelic expression in most humans [52]. At the murine *Kcnq1* domain, the Kcnq1ot1 lncRNA expression brings about allelic repression at many genes in the placenta. In human trophoblastic cells, however, several of these genes seem not to be imprinted [171]. Another issue relative to human studies is limited tissue availability and a lack of single nucleotide polymorphisms to distinguish the parental chromosomes. Nevertheless, these are exciting times, with frequent new discoveries from clinical and mouse studies. During the coming years, undoubtedly, further insights into the regulation and roles of imprinted lncRNAs will be obtained, as well as how they contribute to development and disease.

**Table 1:** Molecular properties of regulatory imprinted lncRNAs in the mouse.

Imprinted Domain	lncRNA Name	lncRNA Gene Location ^1^	Transcript(s) Size (nt) ^2^	Expressed Allele	Action	lncRNA Function and Molecular Mechanism
*Dlk1-Dio3*	Meg3	Chr12: 109506879-109538163	31,285 (unspliced), 11,488 (v1), 11,476 (v2), 1924 (v3)	Maternal	*Cis*	Silencing of *Dlk1*, probably by PRC2 scaffolding and histone methylation deposition [78,84]
					*Trans*	Enhances *Hox* gene repression by H3K27me3, by facilitating the interaction between EZH2 (PRC2 complex) and JARID2 [152]
						In humans, activation of a p53 target gene subset by an unknown mechanism [158]
						In humans, activation of a TGF-beta pathway target gene subset by formation of RNA–DNA triplex structures at distal regulatory elements [145]
*Igf2-H19*	H19	Chr7: 142129267-142131883	2625 (unspliced), 2288 (v1, spliced), 2284 (v2, spliced)	Maternal	*Trans*	Influences an imprinted gene network (including *Igf2*) by MBD1 recruitment and subsequent histone KMT interaction at specific genes [142]
						Promotes decaying of unstable mRNAs through interaction with the KSRP protein [159]
						Hosting and processing regulation of the microRNA precursor miR-675 to control *Igf1r* expression in placenta [41]
						In humans, functions as a tumour suppressor through 4E-BP1 binding and mTORC1 inhibition in pituitary tumours [172]
*Kcnq1*	Kcnq1ot1	Chr7: 142766848-142850284	>83,437 (unspliced)	Paternal	*Cis*	Silencing of *Kcnq1*, *Cdkn1c*, *Slc22a18*, and *Phlda2* in the embryo and the placenta [68,74,75] Silencing of the *Ascl2, Cd81, Tssc4,* and *Osbpl5* genes in the placenta through recruitment of PRC complexes and KMT EHMT2 [50,68,72,74,126]
					*Trans*	In human cells, this lncRNA contributes to retrotransposon repression by influencing HP1 binding [149]
*Igf2r*	Airn	Chr17: 12960198-13079023	118,574 (unspliced), 1176 (v1), 413 (v2), 604 (v3), 1399 (v4)	Paternal	*Cis*	Silencing of *Igf2r* through transcriptional interference [47]
						Silencing of *Slc222a3* and several other genes in the placenta through recruitment of PRC complexes and KMT EHMT2 [46,49,50,51]
*Gnas*	Nespas	Chr2: 174123030-174137229, complement	14,200 (unspliced), 2248 (v1)	Paternal	*Cis*	Silencing of *Nesp*, likely through a transcription-mediated process [60,107]
					*Trans*	Modulation of *IKBKE* and *Tmed9* expression levels by hosting the miR-296 microRNA [162]
*Snrpn*	Snhg14 (Ube3a-ATS)	Chr7: 58922485-60099925, complement	117,7441 (unspliced), 24,206 (v1) and >13 variants with diff. 5′ and 3′ ends	Paternal	*Cis*	Regulation of *Ube3a* expression by transcriptional interference [95,104]
	IPW ^3^	Chr15: 25116545-25122476	5932 (unspliced), 4498 (v1)	Paternal	*Trans*	In humans, downregulation of the Meg3 ncRNA polycistron by mediating repressive histone methylation [143]

^1^ Mouse GRCm39 assembly. ^2^ As annotated at the gene database from the NIH National Library of Medicine. ^3^ lncRNA name, chromosome location, and splice variants according to the human CHCh38.p14 assembly.

## Data Availability

Not applicable.

## Abbreviations

AS: Angelman Syndrome; BWS: Beckwith-Wiedemann Syndrome; DMR: differentially methylated region; FISH: fluorescence in situ hybridisation; ICR: imprinting control region; KMT: lysine methyl transferase; KOS14: Kagami-Ogata Syndrome; lncRNA: long non-coding RNA; LQ1: long QT syndrome type 1; mRNA: messenger RNA; miRNA: microRNA; ncRNA: non-coding RNA; NPC: neuronal progenitor cell; PRC: Polycomb repressive complex; RNA PolII: RNA polymerase II; PHP: pseudo-hypoparathyroidism; PWS: Prader-Willi Syndrome; SRS: Silver-Russell Syndrome; TS14: Temple Syndrome.
